# Morphology-driven downscaling of *Streptomyces lividans* to micro-cultivation

**DOI:** 10.1007/s10482-017-0967-7

**Published:** 2017-11-01

**Authors:** Dino van Dissel, Gilles P. van Wezel

**Affiliations:** Molecular Biotechnology, Institute of Biology, Leiden University, PO Box 9505, 2300RA Leiden, The Netherlands

**Keywords:** High-throughput screening, Micro-cultivation, Morphology, Antibiotic, Enzyme, Actinobacteria

## Abstract

Actinobacteria are prolific producers of secondary metabolites and industrially relevant enzymes. Growth of these mycelial micro-organisms in small culture volumes is challenging due to their complex morphology. Since morphology and production are typically linked, scaling down culture volumes requires better control over morphogenesis. In larger scale platforms, ranging from shake flasks to bioreactors, the hydrodynamics play an important role in shaping the morphology and determining product formation. Here, we report on the effects of agitation on the mycelial morphology of *Streptomyces lividans* grown in microtitre plates. Our work shows that at the appropriate agitation rates cultures can be scaled down to volumes as small as 100 µl while maintaining the same morphology as seen in larger scale platforms. Using image analysis and principal component analysis we compared the morphologies of the cultures; when agitated at 1400–1600 rpm the mycelial morphology in micro-cultures was similar to that obtained in shake flasks, while product formation was also maintained. Our study shows that the morphology of actinobacteria in micro-cultures can be controlled in a similar manner as in larger scale cultures by carefully controlling the mixing rate. This could facilitate high-throughput screening and upscaling.

**Electronic supplementary material:**

The online version of this article (10.1007/s10482-017-0967-7) contains supplementary material, which is available to authorized users.

## Introduction

Actinobacteria produce a plethora of bioactive natural products, such as antibiotics, anticancer agents, immunosuppressants and antifungals (Barka et al. 2016; Bérdy 2005; Hopwood 2007). In addition, these bacteria produce many industrially relevant enzymes, such as cellulases, amylases and proteases (Vrancken and Anne 2009). Streptomycetes exhibit a complex multicellular life cycle (Claessen et al. 2014). This starts with a single spore that germinates to form vegetative hyphae, which then grow out following a process of hyphal growth and branching to produce a branched vegetative mycelium (Chater and Losick 1997). Nutrient depletion and other environmental stresses induce development, whereby aerial hyphae are formed that differentiate into chains of spores following a complex cell division event whereby ladders of septa are produced within a short time span (Jakimowicz and van Wezel 2012; McCormick 2009). The developmental cycle also influences the vegetative mycelia, which partially undergoes programmed cell death to liberate nutrients (Manteca et al. 2005), and chemical differentiation leading to the production of antibiotics and other specialized metabolites (van Wezel and McDowall 2011).

In a submerged environment streptomycetes grow as mycelial networks, typically forming large pellets or clumps. From the industrial perspective, growth as pellets is unattractive, in particular because of mass-transfer problems, slow growth and culture heterogeneity (van Dissel et al. 2014; van Wezel et al. 2009). For antibiotic production pellets are generally beneficial, most likely by promoting pathways linked to stress, nutrient limitation and PCD (van Wezel et al. 2006; Martin and Bushell 1996; Manteca et al. 2008).

High throughput (HT) cultivation methods at a small scale are highly desirable, among others to exploit the potential of newly isolated actinobacteria (Kolter and van Wezel 2016). Down-scaling of culture volumes, while maintaining key factors that influence the productivity seen in shake flasks or small scale bioreactors, is necessary to make large screening efforts rapid and economically feasible (Long et al. 2014). However, growing streptomycetes in small cultures is challenging. Streptomycetes typically display a wide range of morphologies in submerged cultures, including dense pellets as well as large mycelial mats [reviewed in van Dissel et al. (2014)]. After inoculation, spores germinate and produce at least two different extracellular polysaccharides (EPS), a cellulose-like polymer catalized by GlxA (Liman et al. 2013; Petrus et al. 2016) and a second EPS synthesized by MatAB (van Dissel et al. 2015). Both polymers induce germling aggregation, and play a key role in pellet formation [Zacchetti et al. (2016) and our unpublished data]. Germling aggregation promotes the formation of pellets, spatially heterogeneous structures with a largely physiologically inactive core, while the peripheral hyphae grow exponentially by tip extension and branching (Celler et al. 2012). The environment also impacts the morphology profoundly. Among others, the composition of the media (Bushell 1988; Glazebrook et al. 1990; Jonsbu et al. 2002), the viscosity (O’Cleirigh et al. 2005) and the pH (Glazebrook et al. 1992) all affect the physiology of the culture, but the hydrodynamics of the culture probably has the most impact (Olmos et al. 2013). Higher agitation reduces pellet size by promoting fragmentation (Belmar-Beiny and Thomas 1991; Reichl et al. 1992). Fragmentation promotes smaller pellets, thereby increasing the overall growth rate, but increased agitation also results in cell lysis. The relationship between pellet morphology, hydrodynamics (and oxygen supply) and production has been well studied for bioreactors (Tamura et al. 1997; Roubos et al. 2001; Ohta et al. 1995) and for shake flasks (Mehmood et al. 2012; Dobson et al. 2008), but not for smaller cultivation platforms.

To successfully down-scale liquid-grown cultures, the morphology *Streptomyces* mycelia adopt in larger scale platforms (i.e. shake flasks or bioreactors) should be mimicked as closely as possible. The exact morphology, determined by size, density and shape, also depends on the characteristics of the environment (Wucherpfennig et al. 2010). The hydrodynamics, in other words the characteristics of the agitated medium, is of particular importance as it influences among others the rate of fragmentation (Olmos et al. 2013). Low agitation causes poor distribution of nutrients and reduced oxygen transfer rates, stunting growth and production, while strong agitation can cause cell death (Roubos et al. 2001). Examples of HT cultivation platform for filamentous microorganisms have been described (Minas et al. 2000; Siebenberg et al. 2010; Sohoni et al. 2012). These authors made use of shaken deep-well plates, which results in higher oxygen transfer rates than in small-volume microtitre plates (MTPs) (Duetz et al. 2000). Alternatively the BioLector system with specialized peddle shaped wells allowed the cultivation of 48 parallel 1-ml cultures (Rohe et al. 2012; Huber et al. 2009), which recently was successfully adapted for growth of streptomycetes (Koepff et al. 2017).

In this work we sought to further scale down *Streptomyces* cultures to 100 µl scale. As hosts we used *Streptomyces lividans*, the preferred enzyme production host (Anné et al. 2012), and the related *Streptomyces coelicolor*, a model streptomycete for the study of development and antibiotic production (Barka et al. 2016). Cultures were scaled down from shake flasks to 100 µl cultures, using a digital vortex to obtain the extensive mixing required to control pellet morphology. Using whole slide image analysis, the mycelia were quantified and compared in terms of size and shape. This allowed further optimization of growth in micro-cultures.

## Materials and methods

### Bacterial strains, plasmids

*S. lividans* 66 (Cruz-Morales et al. 2013) was used for morphological analysis and enzyme production and *S. coelicolor* A3(2) M145 was used for antibiotic production. Plasmid pIJ703, which carries the *melC1* and *melC2* genes for heterologous tyrosinase production (Katz et al. 1983), was transformed to its host by protoplast transformation (Kieser et al. 2000). Spores were harvested from soy flour mannitol agar plates and stored in 20% glycerol at −20 °C as described (Kieser et al. 2000). The spore titre was determined by plating serial dilutions on SFM agar plates and counting CFUs.

### Cultivation conditions

For cultivation in shake flasks, *S. lividans* was grown in 30 ml tryptic soy broth (Difco) with 10% sucrose (TSBS) in a 100 ml Erlenmeyer flasks equipped with a stainless steel spring. The flask was inoculated with 10^6^ CFUs/ml and cultivated at 30 °C in an orbital shaker with 1 in. orbit (New Brunswick) at 200 RPM. For the production of tyrosinase 25 µM CuCl_2_ was added to the TSBS medium. For antibiotic production *S. coelicolor* was cultivated in defined glutamate/glucose based mineral media prepared according to (Wentzel et al. 2012). 100 µL media with 10^6^ cfu/ml spores was added to wells of a V-bottom 96 well MTP (Greiner Bio-One, Germany). To minimize evaporation, the plate was covered with a custom moulded silicone sheet made from MoldMax40 (Materion, USA), using the 96 well plate as a mold and included centred aeration holes with a 2 mm diameter for each well. An AeraSeal film (Excel Scientific, USA) was added to the top for sterility, while allowing gas exchange. The combined silicone sheet and AeraSeal film were fastened to the plate using masking tape. A Microplate Genie Digital (Scientific Industries, USA) was used for agitation. This microtitre plate vortex has an orbit of 1 mm with accurate speed control. The rotation speed was confirmed using a Voltcraft DT-10L digital tachometer (Conrad, Germany). The entire setup was placed in a humidity-controlled incubator set to 70% RH and 30 °C. The evaporation rate was around 8 µl per well per day. Each condition was performed three times and the shake flask data consist of five independent experiments.

### Image and data analysis

Image analysis was performed as described by whole slide imaging combined with automated image analysis using imageJ (Willemse et al. 2017). In short, 100 µl sample was transferred to a glass microscope slide and covered by a 24 × 60 cover slip. The slide was mounted in an Axio Observer (Zeiss, Germany) equipped with an automated XY-stage, which allowed whole slide imaging using a 10 × objective. The imageJ plugin for automated image analysis optimized for *Streptomyces* liquid morphology was used to measure particle objects for 12 different features [see ImageJ documentation for mathematical description and (Papagianni 2006) for box surface dimension (BSD) and box mass dimension (BMD)]. Incorrectly analysed pellets (e.g. out-of-focus mycelia) were removed manually. Further data processing was done using Python (Anaconda 4.4.0 distribution). Principal component analysis (PCA) and 2D clustering of the data by a Gaussian mixture model were executed with the included methods in the Scikit-Learn toolbox. 95% confidence interval (CI) was calculated as: 1.96 × std × *n* ^−0.5^. Statistical analysis was performed with the StatsModels Python module using a Tukey’s HSD test, with a family-wise error rate set to 0.05, to assess significant similarities between populations.

### Tyrosinase activity measurement

Tyrosinase activity was measured by the conversion over time l-3,4-dihydroxyphenylalanine spectrophotometrically at a wavelength of 475 nm, as described (van Wezel et al. 2006).

### Actinorhodin quantification

The production of actinorhodin by *S. coelicolor* was determined as follows. Culture supernatant (40 µl) was treated with 0.5 μl 5 M HCl to pH 2–3, extracted with a 0.5 volume of methanol–chloroform (1:1), and centrifuged at 5000 rpm for 10 min. The concentration was calculated from the *A*_542_ (ε542, 18,600) (van Wezel et al. 2006).

## Results

### The morphology of *S. lividans* in shake flasks

**Fig. 1:**
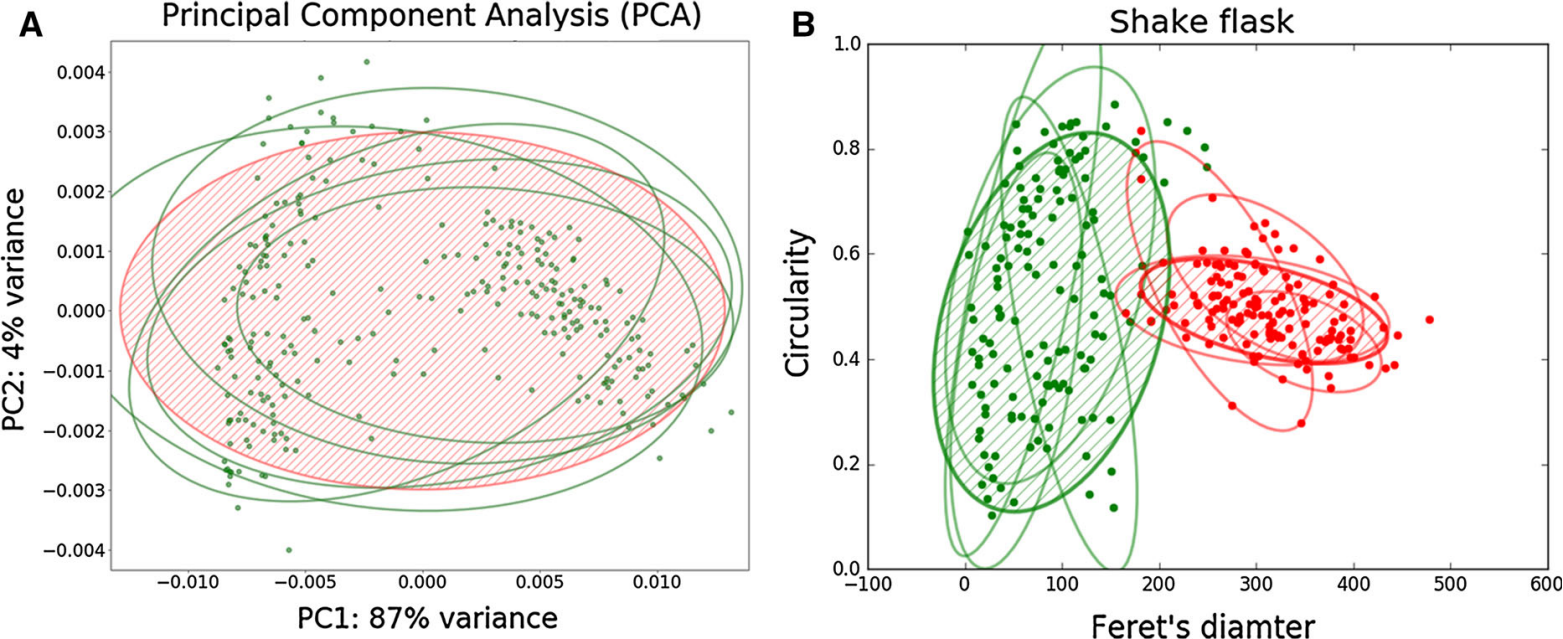
Multivariate analysis of image analysis data from shake flask cultures. **a** Principal component analysis; ellipsoids represent 95% of the data of five independent shake flask cultures and the red area represents the 95% of all combined data. **b** Plot of Feret’s diameter and circularity, which shows the two distinct clusters of pellets found in a heterogeneous culture

To scale down the culture volume, while retaining the morphology, we aimed at replicating key morphological parameters of the liquid-based growth of *S. lividans* in a shake flask, such as pellet formation and fragmentation. We applied the SParticle plugin for ImageJ to quantify the pellets via whole-slide image analysis (Willemse et al. 2017). This plugin allows each particle object to be characterized in 12 different features, allowing multivariate analysis. As a reference, the morphological characteristics of shake flask cultures were investigated. Around 500 aggregates were analysed from five separate 24 h shake flask-grown cultures, corresponding to the end of the exponential growth phase, which roughly corresponds to the moment of antibiotic production initiation (Nieselt et al. 2010). The image analysis data obtained from the shake-flask cultures was PCA transformed to find the features that showed the highest variance (Fig. 1a). This revealed that PC 1 was mainly compromised of features that described the size of the pellets and PC 2 consisted of measurements for its circularity (Table S1). Plotting the Feret’s diameter versus the circularity indeed shows a nice spread of the data points (Fig. 1b). Previous work comparing the maximum length of pellets revealed two different mycelial populations of *S. lividans*, one forming larger and one smaller pellets (van Veluw et al. 2012). To separate these two populations in both diameter and circularity, the data was clustered using a Gaussian mixture model, creating an accurate description of the morphology. This indeed revealed two distinct clusters of particles that not only differ in size, but also in shape (Fig. 1b). One population (left, green cluster in Fig. 1b) had pellets with similar Feret’s diameter of around 87 µm (± 10%), but with a wide standard deviation in circularity (Fig. 1b, green dots and ellipse). Pellets in the second cluster (right, red cluster in Fig. 1b) consisted of larger pellets with an average of 313 µm (± 3%) and were more regularly shaped (Fig. 1b, red dots and ellipse). Because of the effects of pellets on production and regulation it is important to capture all of these morphological characteristics when scaling down.

### Influence of agitation rate on pellet morphology

The morphology of liquid-grown cultures of filamentous micro-organisms is a complex process, whereby hydrodynamics play an important role. These determine the shear stress to which the micro-organisms are subjected, affecting mycelial aggregation and fragmentation, besides also regulating the k_L_a, a measure for the rate of gas exchange. The importance of sufficient mixing for filamentous micro-organisms is exemplified by the need to add a spring or glass beads to a shake flask, which increases the shear stress and promotes growth (Doull and Vining 1989).

We hypothesized that many problems with cultivation of filamentous micro-organisms in small volumes are the result of improper mixing. A digital vortex, designed for microtitre plate (MTP) mixing, allows much higher mixing rates than normally tested and its variable speed settings allow the study of morphology in relation to the agitation rate. This was used to establish whether (and at what agitation rate) a population could be obtained with morphological characteristics similar to those found in larger scale cultures.

**Fig. 2:**
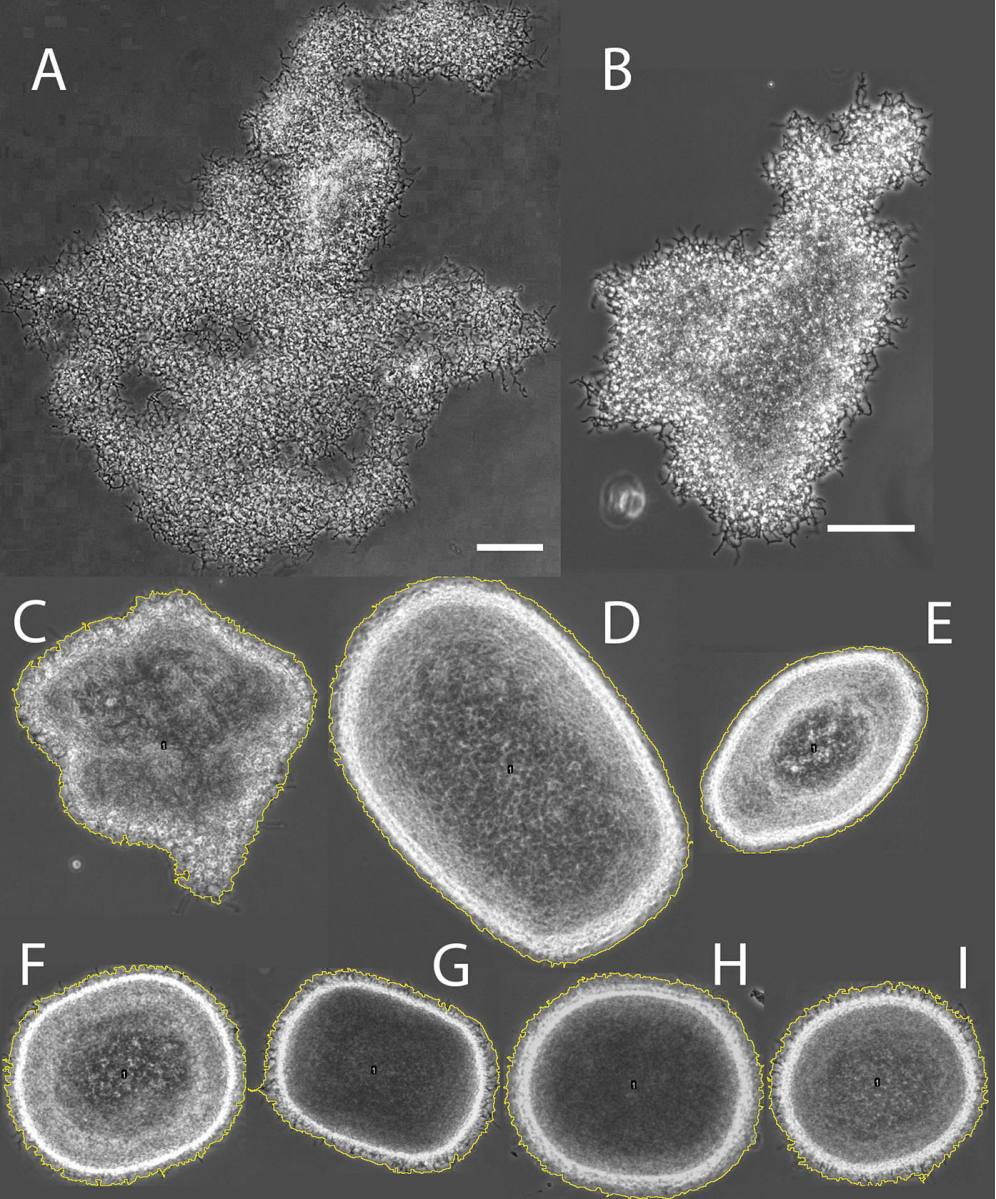
Representative micrograph of mycelial aggregates found around the centroid of the right cluster, for each cultivation condition. **a** Standing culture; **b**–**i** MTP cultures shaken at **b** 600 rpm, **c** 800 rpm; **d** 1000 rpm; **e** 1200 rpm; **f** 1400 rpm; **g** 1600 rpm; **h** 1800 rpm; and **i** shake flask. Yellow perimeter signifies the measured object by image analysis. Scale bar: 100 µm and is shared for B-I. (Color figure online)

Standing cultures of *S. lividans* display a dispersed morphology, which is often entangled with neighbours creating large mats of up to millimeter scale, making them difficult to quantify by automated image analysis (Fig. 2a). When the MTP is agitated at the relative low rate of 600 rpm these large mats are still observed, although hyphal aggregation was already seen (Fig. 2b). This trend continued from 800 to 1000 rpm (Fig. 2c, d) where the aggregates became more pellet-like, although still with irregular shapes and large average pellet size as compared to the flask-grown pellets (Fig. 2i). At 1200 rpm the average pellet size decreased further (Fig. 2e), and at 1400 rpm and higher rates (Fig. 2f, h) we could not visually distinguish the pellets from those obtained from flask-grown cultures (Fig. 2i).

**Fig. 3:**
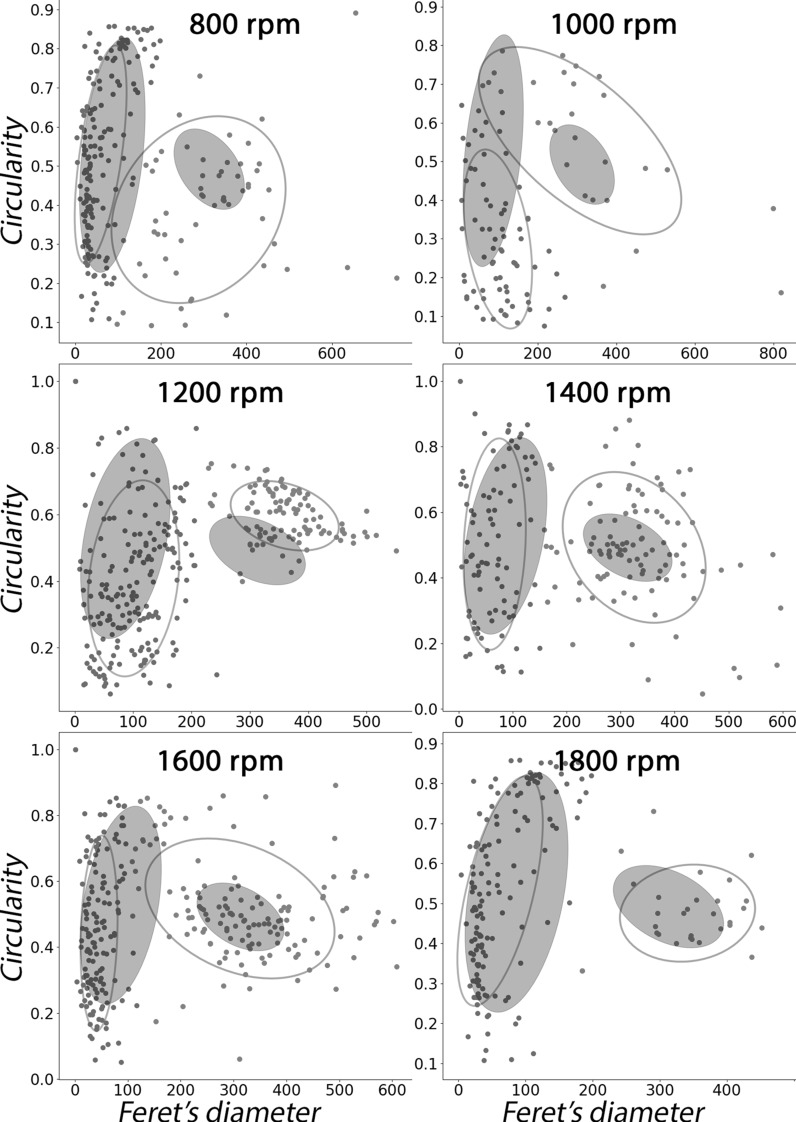
Comparison of micro-cultures with shake flasks by clustering of bi-gaussian population. Feret’s diameter and circularity were plotted for 800, 1000, 1200, 1400, 1600 and 1800 rpm and clustered into two population with a Gaussian mixture model revealing a population of small (green) and large (red) pellets. Blue ellipsoids represent the average populations found for shake flasks. (Color figure online)

**Fig. 4:**
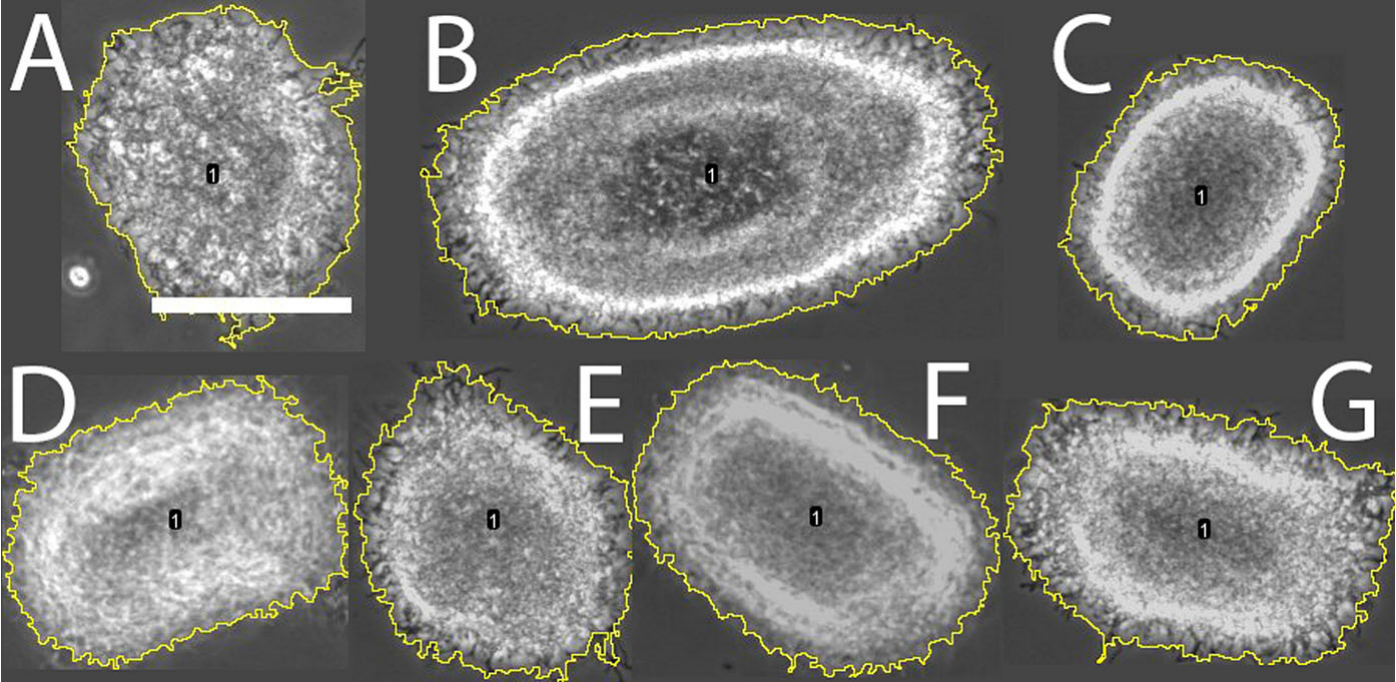
Representative micrograph of mycelial aggregates found around the centroid of the left cluster, for each cultivation condition. **a** 800 rpm; **b** 1000 rpm; **c** 1200 rpm; **d** 1400 rpm; **e** 1600 rpm; **f** 1800 rpm and **g** shake flask. Yellow perimeter signifies the measured object by image analysis. Scale bar: 100 µm. (Color figure online)

To describe the differences quantitatively, the Feret’s diameter and circularity were plotted and clustered to compare them to the shake flask data (Fig. 3; Table 1). This confirmed that pellets obtained from MTP plates shaken at agitation rates of 800 rpm and 1000 rpm share little similarity to those from shake flask cultures, while at higher agitation rates the features are very similar. Where the population of small pellets from a shake flask had an average diameter of 85 µm (± 10%) (left and green in Fig. 1 and Fig. 4g), small pellets in MTP cultures grown at 1200 rpm to 1400 rpm had similar averages (101 µm (± 7%) and 67 µm (± 12%) respectively) (Fig. 4c, d). The sizes of the population of larger pellets also compared well, with an average diameter of 326 µm (± 5%) and 312 µm (± 6%), for 1400 and 1600 rpm, respectively, against 313 µm (± 3%) for shake flasks. The average pellet circularity was also very similar between shake flask-grown cultures and MTP-grown pellets at 1400–1800 rpm, although higher variability was seen in MTP-grown pellets (Fig. 3). To statistically assess the similarity, the 12 features measured by image analysis were used to compare each agitation condition with the shake flask data by a Tukey’s HSD test (Table 2). Concerning the Feret’s diameter and circularity, pellets were similar at 1200–1600, with 1400 and 1600 rpm showing highest similarity when all features are taken into account.

**Table 1:** Length and circularity of the populations of mycelial aggregates under different growth conditions

Culture	Feature	µ1 (µm)	95% CI (µ1, µm)	µ2 (µm)	95% CI (µ2, µm)	PF1^a^ (%)
Shake flask	Feret’s diameter	87	78–95	313	303–323	51
	Circularity	0.53	0.49–0.56	0.49	0.48–0.50	
800 rpm	Feret’s diameter	59	54–65	288	255–321	75
	Circularity	0.53	0.50–0.56	0.39	0.35–0.43	
1000 rpm	Feret’s diameter	99	84–113	312	245–378	28
	Circularity	0.30	0–0.34	0.55	0.49–0.62	
1200 rpm	Feret’s diameter	101	94–108	360	348–372	65
	Circularity	0.41	0–0.44	0.60	0.58–0.61	
1400 rpm	Feret’s diameter	67	59–75	326	309–343	47
	Circularity	0.50	0.46–0.55	0.49	0.46–0.52	
1600 rpm	Feret’s diameter	46	42–50	312	293–332	49
	Circularity	0.44	0.41–0.48	0.52	0.49–0.54	
1800 rpm	Feret’s diameter	62	56–69	341	317–366	86
	Circularity	0.53	0.50–0.56	0.48	0.44–0.51	

*CI* 95% confidence level

^a^PF1 is the participation factor of cluster 1 to the entire population

**Table 2:** Tukey’s HSD test on each of 12 different image analysis features

Comparison		Significant similarity in feature													
		Area	Circ	Max Feret	Perimeter		Round	Mean	SD	Min Feret	Ellipse perimeter	BMD	BSD	Perimeter ratio	Total
800 rpm	SF	+	−	+	+		+	−	+	+	+	−	+	−	8
1000 rpm	SF	−	−	−	−		−	+	−	−	−	−	+	−	2
1200 rpm	SF	+	+	+	−		−	−	+	−	+	+	+	−	8
1400 rpm	SF	+	+	+	+		+	−	+	+	+	+	+	+	11
1600 rpm	SF	+	+	+	+		+	−	+	+	+	+	−	+	10
1800 rpm	SF	−	+	−	−		−	−	−	−	−	−	−	−	1

**Fig. 5:**
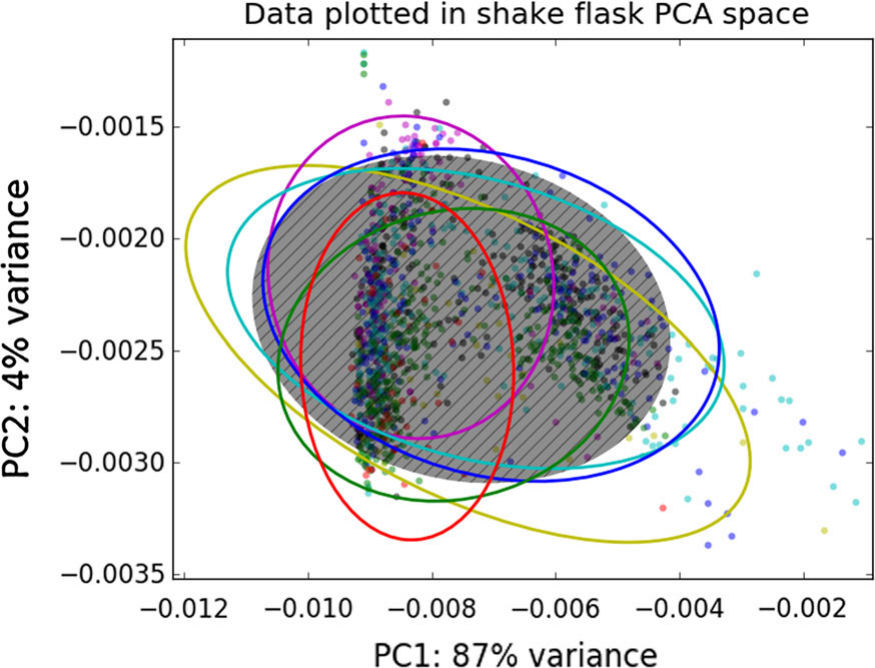
Comparison of pellets from micro-cultures and shake flask cultivations by PCA. Image analysis data obtained from the micro-cultures transformed into the PCA space obtained from the shake flask data. Ellipses represent 95% of the data. Yellow: 800 rpm; red: 1000 rpm; green: 1200 rpm; blue: 1400 rpm; cyan: 1600 rpm; magenta: 1800 rpm; black: Shake flask. (Color figure online)

The PCA space calculated for the shake flask data represented the space with the highest variability (Fig. 1a) and hence this was used to analyse MTP-grown cultures for comparison. For this purpose, all data was transformed into the PCA space calculated for the shake flask data (Fig. 5). The transformed data was subjected to a Tukey test for both principal components (Table 3). This again demonstrated that pellets obtained from 100 µl MTP cultures agitated at 1400 or 1600 rpm are morphologically very similar to those from shake flask-grown cultures.

**Table 3:** Tukey’s HDS test on MPT data PCA transformed in the shake flask space

Comparison		Significant similarity in feature							
		PC1				PC2			
		Mean diff	Lower	Upper	Accept	Mean diff	Lower	Upper	Accept
800 rpm	SF	1.0E−03	−7.0E−04	1.1E−03	−	3.0E−04	0.0E+00	4.0E−04	−
1000 rpm	SF	4.0E−04	4.0E−04	1.7E−03	−	2.0E−04	1.0E−04	4.0E−04	−
1200 rpm	SF	−2.0E−04	0.0E+00	9.0E−04	+	1.0E−04	1.0E−04	1.0E−04	−
1400 rpm	SF	1.0E−04	−7.0E−04	4.0E−04	+	1.0E−04	−1.0E−04	2.0E−04	+
1600 rpm	SF	1.1E−03	1.0E−04	−4.0E−04	+	−1.0E−04	0.0E+00	2.0E−04	+
1800 rpm	SF	2.0E−04	1.1E−03	6.0E−04	−	2.0E−04	−2.0E−04	0.0E+00	−

### Production of heterologous enzymes and antibiotics

**Fig. 6:**
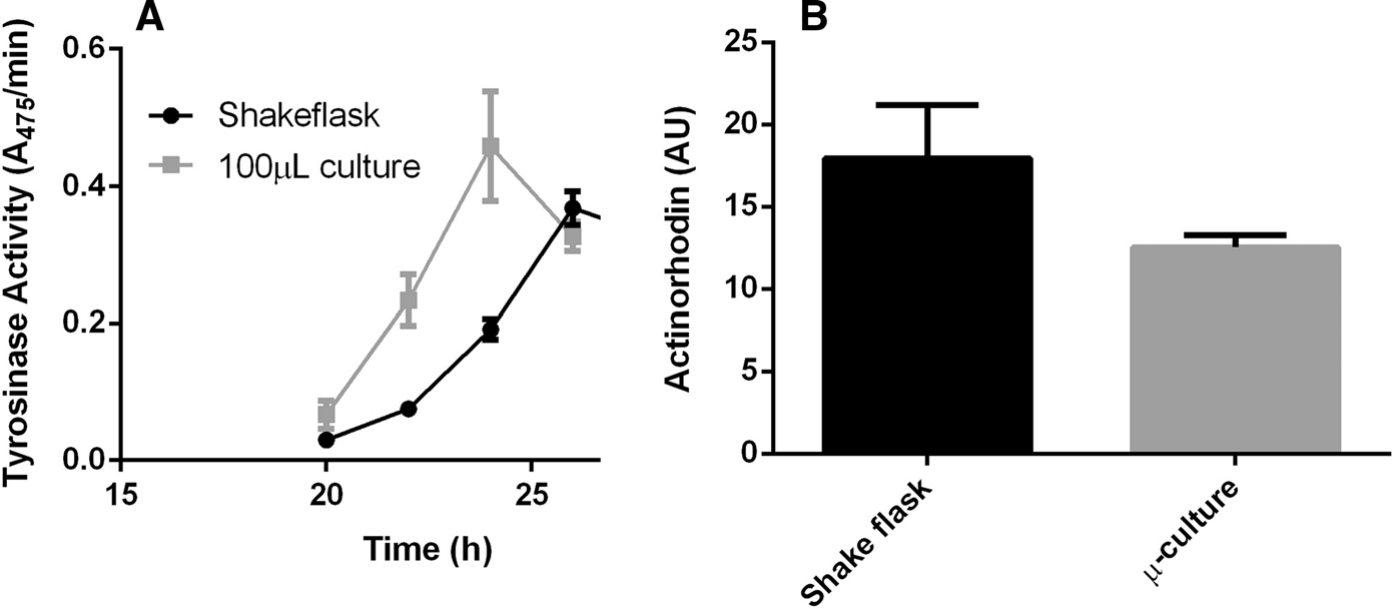
Comparison of enzyme and antibiotic production. **a** Tyrosinase production by *S. lividans* 66. Transformants of *S. lividans* 66 heterologously expressing the secreted enzyme tyrosinase from plasmid pIJ703 were grown in TSBS in either shake flasks or V-bottom MTPs. The graph represents the conversion rate of l-3,4-dihydroxy-phenylalanine by the culture supernatant, which is indicative of tyrosinase activity. Experiments with shake flasks were done in duplicate, while triplicates were used for MTP-grown cultures. **b** Actinorhodin production by *S. coelicolor* M145. *S. coelicolor* M145 was cultivated in minimal media for 48 h. The shake flasks were run in duplicate, while antibiotic production was measured in three different wells in the MTP. Actinorhodin was extracted by chloroform/methanol and measured spectrophotometrically at 542 nm. The average amount of actinorhodin (in arbitrary units) concentration and the standard error of mean of three independent cultures are shown

The above data indicate that 24 h old mycelia obtained from MTP cultures and 1400 rpm agitation are similar to those from shake flasks. To obtain insights into how the producing capacity of the two types of cultures compared, we performed pilot experiments on enzyme and antibiotic production. As model system for extracellular enzyme production we used tyrosinase, heterologously expressed in *S. lividans* by the introduction of plasmid pIJ703 (van Wezel et al. 2006). In line with the similar morphologies, a similar amount of active enzyme was produced in shake flasks (200 rpm, 1 in. orbital) and in MTPs (1400 rpm, 1 mm orbital), although production started slightly earlier in MTPs (Fig. 6a). As model for antibiotic production *S. coelicolor* M145 was used. This strain produces the blue-pigmented polyketide antibiotic actinorhodin, which is readily assessed spectrophotometrically. We compared the growth and production of actinorhodin between shake flasks and micro-cultures grown at 1400 rpm. Similar to MTP cultivations with *S. lividans,* 100 µl cultures grown at 1400 rpm were found to be significantly similar by image analysis (Table S2; Fig S1). After 48 h of growth production of actinorhodin was about 30% higher in shake flasks than in the microcultures (Fig. 6b). Actinorhodin is a stress-related molecule and its production is that shows strong growth phase-dependence. This may explain the slight differences in timing and stress perceived by the mycelia under the different conditions. Taken together, the enzyme and antibiotic assays indicate that production of this antibiotic is comparable between the two culturing methods.

## Discussion

High-throughput screening of actinobacteria for natural products or enzymes typically takes place in micro-scale liquid-grown cultures in an MTP-based setup. The alternative is solid-grown cultures, but it is very difficult to translate growth conditions from solid- to liquid-grown cultures. A drawback of screening of actinobacteria in submerged cultures is the formation of large mycelial networks, which show flocculation or attachment to abiotic surfaces and are associated with slow growth (van Dissel et al. 2014). Additionally, cultures tend to be highly heterogeneous due to the large surface area of the mycelial clumps. Recently, we showed that aggregation of germlings dictates culture heterogeneity (Zacchetti et al. 2016). Because heterogeneity creates a distribution of morphologies, all contributing to production differently (van Veluw et al. 2012; Martin and Bushell 1996), a large population is often required to maintain reproducibility. As heterogeneity is influenced by environmental parameters, careful control is needed to mimic the morphology of a shake flask in small-scale cultivation platform.

Genetically engineered strains have been developed that result in dispersed growth, via over-expression of the cell division activator gene *ssgA* or deletion of the *mat* genes that specify the production of an extracellular polysaccharide involved in hyphal aggregation (van Wezel et al. 2006; Traag and Wezel 2008; van Dissel et al. 2015). However, such genetic manipulation may have major consequences for growth and production. Experiments with *S. coelicolor* grown in 1 ml cultures in deep well plates shaken at 300 rpm revealed that while the produced amount was similar as to shake flasks, the growth and actinorhodin production rate were reduced (Minas et al. 2000). However, the morphology was not monitored in this study, so it is unclear if a suboptimal morphology was the reason for the reduced productivity. Growth in 24-square deep well microtitre plates, shaken at 150 rpm, required a complex pre-culture procedure and the addition of glass beads, to obtain reproducible growth, and although growth and production rates were comparable to a bioreactor, the morphology of the organism was completely different, which could cause problems with upscaling later (Sohoni et al. 2012). Recently, the BioLector system was successfully adapted for growth and screening of streptomycetes, which allows parallel growth of 48 cultures in a MTP with around 1 ml volume (Koepff et al. 2017). This system uses specialized peddle-shaped plates for cultivation, and allows the monitoring of OD, pH and DO. The authors obtained promising results, obtaining growth parameters that could be compared to those seen in 1 l cultures.

In this study, we show that streptomycetes can be successfully cultivated even in 100 µl micro-cultures, without the use of specialized equipment or extended pre-culture procedures, while maintaining the same morphology as in large shake flasks. Our data show that the distribution of a heterogeneous mycelial population is highly dependent on the agitation rate in 96-well MTPs. Under the conditions chosen, pellets obtained from 100 µl MTP cultures agitated at 1400 or 1600 rpm were morphologically highly comparable to those obtained from shake flask-grown cultures, as shown by image analysis. An initial comparison of the productivity between shake flasks and microcultures revealed comparable yields for the enzyme tyrosinase and the antibiotic actinorhodin produced by *S. lividans* 66 and *S. coelicolor* M145 respectively. More extensive studies are required to assess the effect of microcultures on productivity. When required, the level and timing of antibiotic production can be changed by altering the promoter of the pathway-specific regulatory gene that controls the biosynthetic gene cluster of interest (Bibb 2005; van Wezel et al. 2000).

At insufficient mixing rates the mycelia failed to aggregate into typical pellet structures. This may at least in part be explained by insufficient oxygen supply. The relationship between oxygen supply and morphology is not well understood, but preliminary experiments where the oxygen supply was limited in a shake flask by reducing the gas exchange, resulted in pellets with a reduced density similar to what was found in poorly agitated MTPs (DvD and GPvW, unpublished results). Although the k_L_a was not measured in this study, initial calculations using equations for orbital mixing (Seletzky et al. 2007) showed that the oxygen transfer could be lower than adequate, with a k_L_a as low as 40 h^−1^, for a mixing rate of 800 rpm. This low value is suggestive of oxygen limitation as the cause of the morphology observed at low agitation rates and that in part the change in morphology by increased agitation is the result of an increased oxygen supply. While these observations are indicative of oxygen limitation as determining factor for mycelial morphology, oxygen transfer and hydrodynamic stress are coupled processes for orbital shaken cultivation methods (and to some extent also for bioreactors). At least for pristinamycin production hydrodynamic stress, described as the power input, was more descriptive for both pellet morphology and production levels (Mehmood et al. 2012). Thus, downscaling to 100 µl is feasible for *Streptomyces*, even if it aggregates into dense pellets. How precisely agitation affects morphogenesis in MTP plates is as yet unclear and requires further study.

Matching the environment, including the physical hydrodynamic forces that determine the morphology is due to its complex nature a difficult task. Our study also illustrates the utility of image analysis to quantify the morphology and assist in the down-scaling process. We devolved a workflow to characterize the morphology of an *Streptomyces* population, which works in conjunction with the recently published imageJ plugin SParticle, an automated image analysis toolbox to study the morphology of liquid-grown *Actinomyces* cultures (Willemse et al. 2017). Prior knowledge of the population dynamics of liquid grown cultures, which could be described by a bi-gaussian distribution of *Streptomyces* particle, was used to characterize shake flask cultures. Comparison of maximal pellet diameter and circularity provides more detailed insights into the exact morphology of the pellets, which aids the down-scaling process. Besides providing the option of medium- to high-throughput screening, the ability to grow *Streptomyces* with a native morphology on a very small scale also allows studies that involve for example the addition of expensive or low abundance chemicals or enzymes.

## Conclusion

The complex morphology displayed by filamentous actinobacteria in liquid-grown cultures greatly influences their productivity. Screening these bacteria for new therapeutic agents in an MTP-based setup without affecting normal growth and morphology would be a major advantage. This is particularly important in the light of upscaling, so as to maximise the chance that productivity is maintained. We have been able to translate growth and morphology from shake flasks to 100 µl micro cultures by carefully tuning the rate of agitation. The resulting growth and average pellet size in standard HTS-compatible MTPs was reproducibly comparable to those in larger scale cultures, which is an important contribution to the state of the art.

## Electronic supplementary material

Below is the link to the electronic supplementary material.

Supplementary material 1 (PDF 963 kb)

## Abbreviations

BMD: Box mass dimension
BSD: Box surface dimension
CFU: Colony forming units
CI: Confidence interval
HTS: High throughput screening
k_L_a: Specific oxygen transfer coefficient
MTP: Microtitre plate
PC: Principal component
PCA: Principal component analysis
Rpm: Rotations per minute
P: Power dissipation
