# Supplementary material for: Morphology-driven downscaling of Streptomyces lividans to micro-cultivation
Source: Antonie Van Leeuwenhoek. 2017 Nov 1;111(3):457–69. doi: 10.1007/s10482-017-0967-7 (PMC5816114; doi:10.1007/s10482-017-0967-7)

Supplemental Information

belonging to the manuscript

Morphology-driven downscaling of *Streptomyces lividans* to micro-cultivation

Dino van Dissel[1] and Gilles P. van Wezel[1,#]

[1]Molecular Biotechnology, Institute of Biology, Leiden University, PO Box 9505, 2300RA, Leiden, The Netherlands;

[#] To whom correspondence should be addressed. Tel: +3171527430; email: g.wezel@biology.leidenuniv.nl

Table S1. PCA weight matrix for shake flask data

Attribute #	Contribution to matrix	
	PC1	PC2
Area	0.55	-0.17
Mean	0.00	-0.22
Stdev	0.20	-0.12
Round	0.19	0.60
Circularity	0.00	0.71
Max Feret	0.35	-0.05
Min Feret	0.43	0.18
Perimeter	0.42	-0.10
Elipse Perimeter	0.36	-0.04
Perimeter ratio	0.11	0.04
BMD	0.01	-0.02
BSD	0.00	0.00

Stdev – standard deviation; BSD - box surface dimension; BMD - box mass dimension; PC – principal component.

Table S2. Tukey's HSD test assessing similarity of *S. coelicolor* grown in a shake flask or an MTP at 1400 rpm.

Comparison		Significant similarity in attribute #													
		Area	Circ	Max Feret	Perimeter	Round	Mean	Stdev	Min Feret	Elipse Perimeter	BMD	BSD	Perimeter Ratio	PC1	PC2
1400 rpm	SF-SCO	+	+	+	+	+	-	+	+	+	+	+	+	+	+

MTP – microtitre plate; Stdev – standard deviation; rpm – rotations per minute; BSD - box surface dimension; BMD - box mass dimension; Circ. – circularity; PC – principal component.

Fig. S1. Representative pellets of *S. coelicolor* M145 in shake flasks and microcultures shaken at 1400 rpm. *S. coelicolor* M145 was grown in TSBS for 24h. Pellets obtained from shake flasks (left) and from microcultivation agitated at 1400 rpm (right) show comparable morphology.

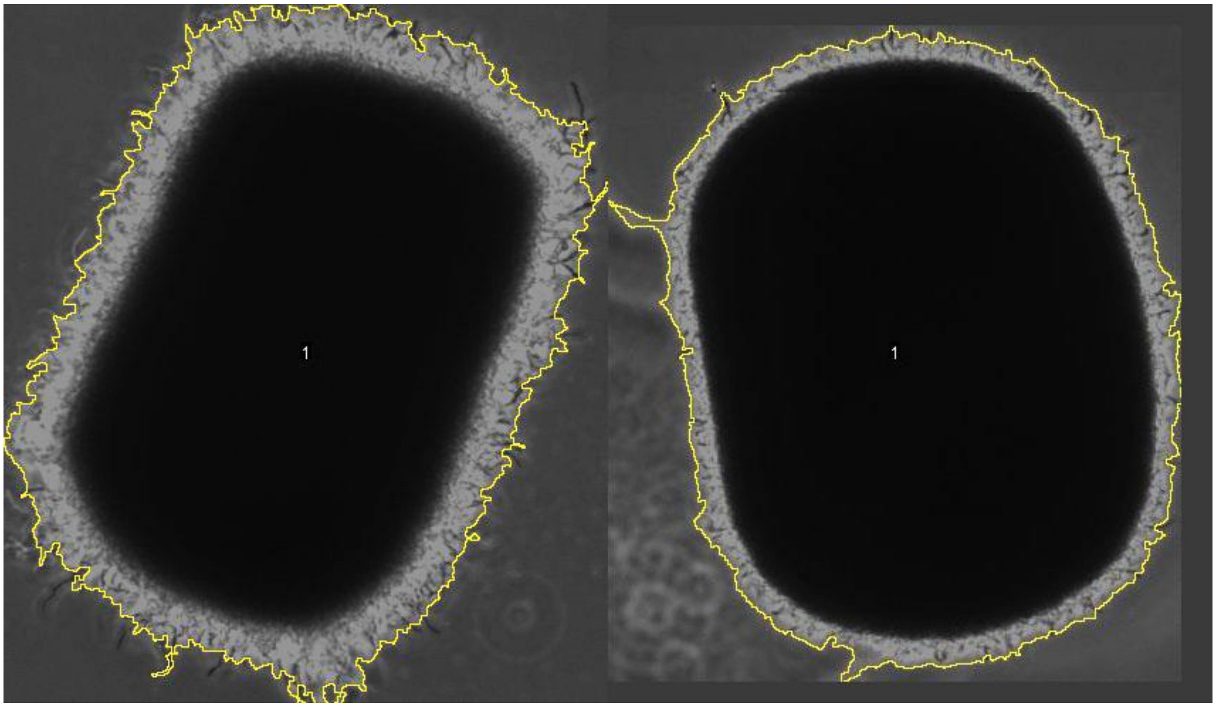

Supplement: Supplementary file 1 — Supplementary material 1 (PDF 963 kb) [file 10482_2017_967_MOESM1_ESM.pdf]
